# Host status and susceptibility of *Cannabis sativa* cultivars to root-knot nematodes

**DOI:** 10.2478/jofnem-2024-0003

**Published:** 2024-03-14

**Authors:** J. Coburn, J. Desaeger

**Affiliations:** University of Florida, Department of Entomology and Nematology, Gulf Coast Research and Education Center, Wimauma, FL 33598, USA

**Keywords:** hemp, *Meloidogyne* spp., cultivars

## Abstract

Root-knot nematode host status of hemp cultivars of different uses (fiber, dual, CBD/CBG) and from different regions (Europe, China, US) were evaluated in five different greenhouse trials. None of the tested cultivars showed resistance to any of the tested root-knot nematode species, and all tested hemp cultivars were good hosts for root-knot nematodes, especially to mixed populations of *M. javanica* and *M. incognita*. Root gall symptoms on hemp were less severe than on cucumber (and tomato), but reproduction rates were similar. Lower infection and reproduction rates were noted for *M. hapla* and *M. enterolobii*, which were probably due to the colder temperatures at the time of the trial, as the same effect was noted for the cucumber control plants. While no negative impact on hemp shoot growth was seen in trials where nematodes were added to pasteurized soil, a significant and visible negative effect on hemp growth was noted when two CBG hemp cultivars were planted in heavily naturally root-knot infested soil. This result indicates that hemp is not only a good host to root-knot nematodes, but also that root-knot can be a limiting factor for hemp production in Florida and other places with high abundance and pressure of root-knot nematodes.

Nematodes, especially root-knot nematodes (*Meloidogyne* spp., RKN) are considered among the main limiting factors to crop production in the world (Sasser, 1980; Sasser and Carter, 1985). Root-knot nematodes are extremely prevalent in Florida because of the subtropical climate and often sandy soils, with multiple species and races and a wide host range that includes grasses, vegetables, and fruits. They are the primary nematode problem in Florida vegetables, causing significant losses in tomatoes, cucurbit, and other vegetables. The main species of vegetables are *Meloidogyne javanica, M. incognita, M. arenaria* and *M. enterolobii* (Desaeger, 2018b). In Florida strawberries, *M. hapla*, the northern root-knot nematode, has started to become more important as well (Desaeger, 2018a). Florida farmers who double-crop after the strawberry season with vegetables, such as watermelon, often find *M. hapla* damage on their second crop (Desaeger, 2018a).

Florida growers are increasingly interested in new alternative crops as many of the traditional crops in Florida, such as citrus, fruiting vegetables, and strawberries, are facing progressively more pressure due to disease issues and increasing competition from abroad. With the removal of hemp (*Cannabis sativa* L., <0.3% delta-9-tetrahydrocannabinol) from the controlled substances list (2018 Farm Bill and 2019 Florida Statute, SB1020), hemp now has the potential to be an alternative crop in Florida.

There are multiple types of hemp for various uses, including fiber, grain, dual-use, and cannabinoid extracts (Cherney and Small, 2016; Fike, 2016). Different parts of the hemp plant are harvested for specific purposes. Fiber hemp varieties are known to have tall, preferably slender stalks and can grow anywhere from 1 to 5 m in height. Clothing, rope, housing materials, compost, and paper are a few items that can be made from just the stalk of the fiber hemp plant (Cherney and Small, 2016). There is great potential for fiber hemp products to be used as eco-friendly alternatives or replacements for construction materials and plastics in the future. Grain hemp cultivars are plants that produce a high density of seed from which a multitude of products can be made, including food items, animal feed, and cosmetics (Cherney and Small, 2016). These plants do not grow as tall as fiber plants, usually averaging 1 to 2 m in height. Dual-use varieties tend to produce a large number of seeds to use for hemp grain products, but they also have a stalk that is suitable for use in fiber hemp production. Producers harvesting hemp grain would also harvest the remaining stems for fiber, and this is the most common example of a dual-use hemp crop (Williams and Mundell, 2015).

Hemp that is cultivated and bred to process flower parts for cannabinoid extracts are usually shorter and have a more brachiate or bushy appearance as compared to fiber and grain. CBD, or cannabidiol, is the non-psychoactive compound in marijuana and hemp, and it closely resembles THC or tetrahydrocannabinol (Atakan, 2012). CBD can be obtained from the plant by extracting the oil from the flower or by burning or vaporizing the dried flower material. CBD products include topicals, edible forms, and smokable products and are used medicinally to curb anxiety, pain, cognitive disorders, and to aid in sleeping (Grinspoon, 2018). Cannabinoids are highly concentrated in the trichomes of the bracts of unfertilized female flowers and are much lower in the root and plant tissue, and at even lower concentrations in hemp pollen and seeds (Williams and Mundell, 2015). CBG, or cannabigerol, is another non-psychoactive cannabinoid found in cannabis. Just like CBD hemp, CBG hemp is bred and cultivated for the unpollinated female flower.

Due to legal constraints, only recently has research on hemp been able to begin. Thus, there is a lack of information and knowledge on cultivars that are more tolerant to environmental stressors, including nematodes. Recently, we reported that hops (*Humulus lupulus*), another new crop to Florida and a member of the same plant family (Cannabaceae) as hemp, is a good host to *M. javanica*, the Javanese root-knot nematode (Desaeger, 2018b). Recent reports from the US and China have also shown that hemp can be a good host to *M. incognita*, *M. enterolobii,* and *M. hapla* (Song et al, 2017; Kotcon et al., 2018; Ren et al, 2020; Bernard et al., 2022).

The main focus of this research was to explore the host status and susceptibility of hemp cultivars of different geographical locations and uses to different species of RKN and potentially identify cultivars that are less susceptible to any of the species of root-knot nematode.

## Materials and methods

The host status and susceptibility of 13 different hemp cultivars to root-knot nematode species was evaluated at the University of Florida Gulf Coast Research and Education Center (GCREC) in Wimauma, FL, in a series of five greenhouse studies between April 2019 and March 2021. Hemp cultivars used included fiber, grain, CBD, and CBG cultivars from different geographic regions (Europe, China, US; Table 1). Trial 1 included six European fiber or dual-use cultivars (both with and without nematode inoculation), trial 2 included the same six cultivars plus three Chinese fiber/dual-use and two US CBD cultivars (only with nematode inoculation), and trial 3 included the three Chinese fiber/dual-use and two US CBD cultivars (both with and without nematode inoculation). In trial 4, two US CBG cultivars were tested in a highly infested local field soil (*M. javanica* and *M. incognita*) with and without nematicide or soil pasteurization. In trial 5, we evaluated a CBD cultivar and a fiber cultivar against a tropical RKN *M. enterolobii* and a temperate RKN *M. hapla*.

**Table 1:** Hemp cultivars evaluated with their given use and geographical origin.

**Cultivar**	**Origin**	**Use**
Cherry Blossom	USA	CBD
Cherry Blossom × T1	USA	CBD
Gold	USA	CBG
Panacea	USA	CBG
Bama	South China	DUAL
Carmagnola	Italy	DUAL
Carmagnola Selezionata	Italy	DUAL
Helena	Serbia	DUAL
Tygra	Poland	DUAL
Yuma-2	South China	DUAL
Eletta Campana	Italy	FIBER
Fibranova	Italy	FIBER
Puma-3	South China	FIBER

Use: Fiber (tall, slender varieties used for materials and textiles), Dual (produce a large number of seed to use for grain products but also have a stalk that is used in fiber hemp production), CBD (cannabidiol, a cannabinoid extract), and CBG (cannabigerol, a cannabinoid extract).

Cultivars were screened (1) in pasteurized soil that was inoculated with a natural field population of RKN containing a mixture of *M. incognita* and *M. javanica* (trials 1,2,3); (2) in non-pasteurized, naturally infested soil (mixture of *M. incognita* and *M. javanica*) with and without a nematicide (trial 4); and (3) in pasteurized soil that was inoculated with either *M. enterolobii* or *M. hapla* (trial 5).

For the greenhouse experiments pertaining to the natural field population (trials 1,2,3), RKN eggs were obtained from infected tomato roots from the GCREC farm. Eggs were extracted from galled tomato roots using the NaOCl method as described by Hussey and Barker (1973). Root-knot nematode populations were identified as *M. incognita* (*Mi*) and *M javanica* (*Mj*) via PCR/RFLP, as outlined by Baidoo et al. (2016), where nematode DNA was digested with NaOH and the mitochondrial DNA was amplified by PCR primers TRNAH/MRH106 and restriction enzymes HinfI dig and MnlI dig.

For the naturally infested soil experiment (trial 4), soil containing *M. javanica* and *M. incognita* was collected from an infested cucumber field. For the selected species experiment (trial 5) with two different species of RKN, *Meloidogyne enterolobii* (*Me*) and *M. hapla* (*Mh*) were obtained from pure cultures that were maintained, respectively, on tomato and strawberry in the greenhouse at the GCREC.

For each trial, hemp seeds were presoaked in distilled water for one hour before being placed in a moisture chamber consisting of paper and plastic zipper-sealed bags where they were kept for four days. Four-day-old, germinated seeds were then planted in 20 cm wide × 19 cm tall clay pots that were filled with steamed field soil. The soil used in the experiment was Myakka fine sand (96% sand, 3% silt, and 1% clay, with a pH of 7.6 and 0.8% organic matter) which was pasteurized at 70°C for 12 h in an SST-15 120v Soil Sterilizer (Pro-Grow Supply Corp., Brooksville, WI, USA). Nematode inoculation was done three days after planting by adding 10,000 RKN eggs in 1-mL aliquots, each containing 2,500 RKN eggs, into four small, 2.5 cm-deep holes surrounding the germinated seed. The cucumber cultivar “Dasher II” was used in all trials as a positive control.

The length of each trial, unless otherwise stated, was 60 days from the day of inoculation, allowing multiple generations of RKN to develop within the roots. Pots were arranged on greenhouse benches in a complete randomized design, watered as needed, and fertilizer was applied once a week with approximately 200 mL of 20-20-20 (NPK) per pot.

Throughout the 60-day duration, height measurements were taken bi-weekly. At the completion of the trial, roots were rated for root galls, root-knot eggs were extracted from the whole root system, root-knot juveniles were extracted from the soil, and plant biomass measurements were taken including dry root and shoot weights for each plant. Plant material was dried for one week at 60° Celsius. Gall ratings were completed by following the RKN rating chart, as outlined by Bridge and Page (1980), where zero indicates no galls and ten indicates severe galling on all roots. Root-knot nematode eggs were extracted using the Hussey and Barker (1973) bleach method from the entire root system, and juveniles (J2) were extracted from a 200-cm^3^ soil sample from each pot by means of a modified Baermann funnel method (Viglierchio and Schmitt, 1983). For trials 3 and 5, a reproduction factor (Rf) was calculated for each cultivar after two months by dividing the final population (Pf), the total number of eggs and juveniles after 60 days, by the initial population (Pi) of 10,000 RKN eggs (Rf=Pf/Pi).

Early infection of J2 within the roots was evaluated in trials 1 and 2 by planting two germinated seeds in the nematode-inoculated pots. One seedling was removed one week after inoculation, and nematodes within the roots were differentially stained with a food dye method (Thies et al., 2002). Plant roots were gently washed free of soil and debris, heated for thirty seconds in 12% red food dye, and then de-stained by exposure to warmed acidified glycerin, leaving the stained juvenile inside the root visible. Juveniles were visually observed and quantified using a stereoscope at 40x magnification. Because of the plant removal, no reproduction factor was calculated for trials 1 and 2. Also, for trial 4, no reproduction factor was calculated as naturally infested field soil was used, and the initial egg inoculum could not be quantified.

Data were analyzed via one-way ANOVA through JMP 15 (SAS Institute Inc., 1988–2021) and differences among treatment means were examined by using Tukey’s HSD at *P* ≤ 0.05.

### Trial 1: Host Potential and Susceptibility of European Fiber and Grain Cultivars to a Mixed Population of *M. javanica* and *M. incognita*

Trial 1 was conducted from April to June 2019 to evaluate the host status of six European hemp cultivars (Helena, Tygra, Fibranova, Eletta Campana, Carmagnola, Carmagnola Selezionata, Table 1) to a mixed field population of *M. incognita* and *M. javanica*. The average greenhouse temperature was 25° C ± 3° C. There were ten pots for each cultivar, five pots were inoculated with 10,000 RKN eggs and five were not inoculated.

### Trial 2: Host Potential of European, Chinese, and US Fiber, Grain, and CBD Cultivars to a Mixed Population of *M. javanica* and *M. incognita*

Trial 2 was conducted from June to August 2019 to evaluate the host status of 11 different European, Chinese, and US cultivars (the same six European cultivars as in trial 1, plus the Chinese fiber/dual cultivars Yuma-2, Puma-3, Bama, and two US CBD cultivars Cherry Blossom and Cherry Blossom × T1, Table 1). Cultivars were evaluated against the same mixed population of RKN as in trial 1. The average greenhouse temperature was 26° C ± 4° C. Nematode inoculation was done as explained previously, and there were no uninoculated pots. There were five replicates of each cultivar.

### Trial 3: Host Potential and Susceptibility of Chinese and US Fiber and CBD Cultivars to a Mixed Population of *M. javanica* and *M. incognita*

Trial 3 was conducted from January to March 2021 to evaluate the host status of three Chinese hemp cultivars (Yuma-2, Bama, Puma-3) and two US CBD cultivars (Cherry Blossom × T1, Cherry Blossom) to the same mixed population of RKN as in trial 1, with ten pots for each cultivar, five inoculated with 10,000 RKN eggs and five uninoculated. The average greenhouse temperature was 20° C ± 2° C.

### Trial 4: Evaluation of two US CBG Cultivars in naturally RKN-Infested Soil, with and without Nematicide

Trial 4 was conducted from early-June to late-July 2020. Two-month-old CBG seedlings (cultivars Gold and Panacea) were evaluated and compared with one-month-old tomato seedlings (cultivar HM1823) in naturally infested soil (*M. incognita* and *M. javanica*). In addition, two other treatments were included: (1) a nematicide was drench-applied at planting (Velum®, a.i fluopyram 41.5%, Bayer Crop Science at 0.48 kg a.i./ha), and (2) soil was pasteurized (steamed) prior to filling pots and planting, as described earlier). The naturally infested field soil was collected from a local cucumber field that suffered severe root-knot damage. The soil was mixed thoroughly prior to filling the pots, and the initial population was measured by taking a 200-cm^3^ subsample from the mixed-infested soil as described previously. The initial population was 725 J2/200-cm^3^ soil. The pots were arranged in a completely randomized design across a greenhouse bench. The average greenhouse temperature was 24° C ± 3° C. There were 15 pots for each cultivar, five untreated, five treated with fluopyram, and five with steamed soil.

### Trial 5: Evaluation of Two Hemp Cultivars Against *M. hapla* and *M. enterolobii*

Trial 5 evaluated the host potential of *M. hapla* and *M. enterolobii* in two hemp cultivars (fiber and CBD) and was conducted between January and April 2021. Average greenhouse temperature was 21° C ± 2° C. *Meloidogyne enterolobii* (*Me*) and *M. hapla (Mh)* eggs were obtained by the bleach method from pure cultures of tomato and strawberry, respectively. Hemp cultivars used were the CBD cultivar Cherry Blossom × T1 and the fiber cultivar Eletta Campana. The same methodology was used as in previous experiments.

## Results

### Trial 1: Host Potential and Susceptibility of European Fiber and Grain Cultivars to a Mixed Population of *M. javanica* and *M. incognita*

**Figure 1:**
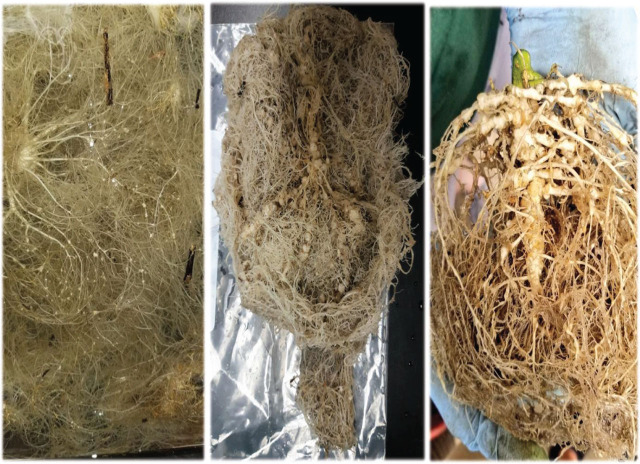
Root galls caused by RKN (*M. javanica* and *M. incognita* mixed population) on hemp roots (cv. Carmagnola Selezionata, left) compared to cucumber roots (cv. Dasher II, middle) (Trial 1) and cv. Cherry Blossom × T1 (right; Trial 2) (Photos J. Coburn).

One week after inoculation, RKN juveniles were found in all hemp cultivars with significantly more J2s in cultivar Carmagnola Selezionata (60 per root) as compared to cultivars Tygra (6) and Helena (15) (*P* = 0.008) (Table 2). For comparison, an average of 45 juveniles per root system were found in cucumber roots. After sixty days, there was no significant difference in root galling among cultivars (average gall index rating of 3.5, *P* = 0.630, compared to a gall rating of 7.0 for cucumber). High RKN reproduction was noted on all six hemp cultivars (up to 523,000 eggs per root for cultivar Tygra), with small but visible root galls and no difference among cultivars (Table 2, Fig. 1).

**Table 2:** Nematode reproduction on six European hemp cultivars after 60 days with a mixed population of *M. incognita* and *M. javanica* (Trial 1).

	**J2/stained root**	**Gall Index (0–10)**	**Eggs/Whole Root**	**Eggs/g of Dry Root**	**J2s/pot**
Helena	15b	2.4	292,020	53,600	600
Tygra	6b	2.6	523,300	138,700	1,500
Fibranova	22ab	2.0	333,600	49,010	1,550
Eletta Campana	45ab	2.6	468,960	56,250	990
Carmagnola	41ab	2.8	401,410	52,420	350
Carmagnola Selezionata	60a	3.0	498,940	71,710	430
Cucumber^*^	45	7.0	460,800	-	225
*P* value	0.008	0.630	0.557	0.501	0.114

J2s/stained root were evaluated after 7 days. Gall index scale: 0–10; 0= no galls, 10= 100% galls. Eggs and J2s rounded to nearest ten. Factor levels not connected by the same letter are significantly different according to Tukey’s HSD with *P* ≤ 0.05; cucumber data are given as comparison and were not included in the analysis.

**Figure 2:**
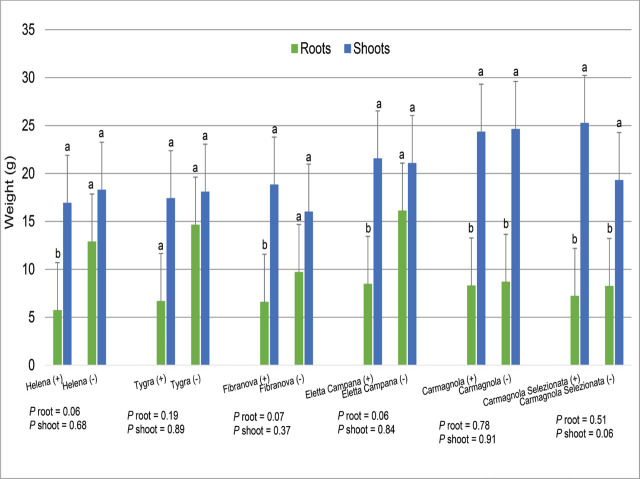
Hemp plant dry weights (g) for inoculated (+) and uninoculated (−) plants with a mixed population of root-knot species and six European hemp cultivars. Factor levels not connected by the same letter are significantly different according to Tukey’s HSD with *P* ≤ 0.05 (Trial 1).

Plant height was similar for all cultivars, ranging between 140 and 170 cm after 60 days, and showed no significant interaction between inoculated and uninoculated plants (data not given). Shoot weight was not negatively affected by nematode inoculation, but root dry weight was slightly reduced in some cultivars such as Tygra, Helena, and Eletta Campana (*P* = 0.065, Fig. 2).

### Trial 2: Host Potential of European, Chinese and US Fiber, Grain, and CBD Cultivars to a Mixed Population of *M. javanica* and *M. incognita*

One week after inoculation, RKN juveniles were found in roots of the Chinese and US cultivars, ranging from 43 to 83 juveniles per root system (no staining was done for other cultivars) (*P* = 0.004, Table 3). Root gall indices were highest for the fiber cultivars (max. GI = 5.8) and lowest for the cultivar Cherry Blossom (GI = 1.5), with the cucumber inoculum check having a root gall index of 7.2 (Table 3). RKN reproduced very well on all 11 hemp cultivars tested, similar to cucumber (up to 270,000 eggs per root for cultivar Fibranova), and no significant difference was noted among cultivars (Table 3).

**Table 3:** Nematode reproduction on 11 hemp cultivars (European, Chinese and American, fiber, dual-use and CBD) after 60 days with a mixed population of *M. incognita* and *M. javanica* (Trial 2).

	**J2/stained root**	**Gall Index (0–10)**	**Eggs/Whole Root**	**Eggs/g of Dry Root**	**J2s/pot**
Helena	-	5.2a	211,100	172,680	19,530ab
Tygra	-	5.8a	107,530	62,640	18,930ab
Fibranova	-	3.8b	271,710	136,540	14,310b
Eletta Campana	-	4.8a	197,730	86,940	23,720a
Carmagnola	-	4.8a	172,330	57,310	17,000b
Carmagnola Selezionata	-	4.8a	101,680	34,650	15,220b
Yuma-2	43	4.4b	270,980	43,590	15,810b
Bama	45	3.4b	153,540	29,740	8,600b
Puma-3	48	3.8b	180,480	31,580	7,420b
Cherry Blossom × T1	83	4.2b	187,900	51,740	11,490b
Cherry Blossom	74	1.8c	68,160	21,320	4,000b
Cucumber^*^	-	7.2	218,260	-	9,702
*P* value	0.01	<0.001	0.599	0.093	0.014

J2s/stained root were evaluated seven days after inoculation. Gall index scale: 0–10; 0= no galls, 10= 100% galls. Eggs and J2s rounded to nearest ten. Factor levels not connected by the same letter are significantly different according to Tukey’s HSD with *P* ≤ 0.05; cucumber data are given as comparison and were not included in the analysis.

**Figure 3:**
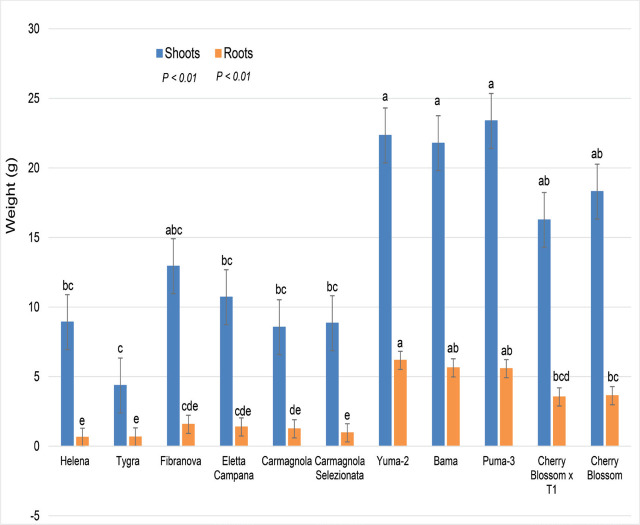
Hemp plant dry weights (g) with a mixed population of root-knot species and 11 hemp cultivars; all plants were inoculated with 10,000 RKN eggs. Factor levels not connected by the same letter are significantly different according to Tukey’s HSD with *P* ≤ 0.05 (Trial 2).

The Chinese fiber and dual-use cultivars had the greatest plant heights, on average 1.5 m after sixty days, European fiber and dual-use cultivars averaged 1 m in height, and the CBD plants averaged just under 1 m (data not given). The same was noted for the plant weights, with cultivars that were of similar uses and geographical origins having similar overall plant biomass (Fig. 3).

### Trial 3: Host Potential and Susceptibility of Chinese Fiber and US CBD Cultivars to a Mixed Population of *M. javanica* and *M. incognita*

**Figure 4:**
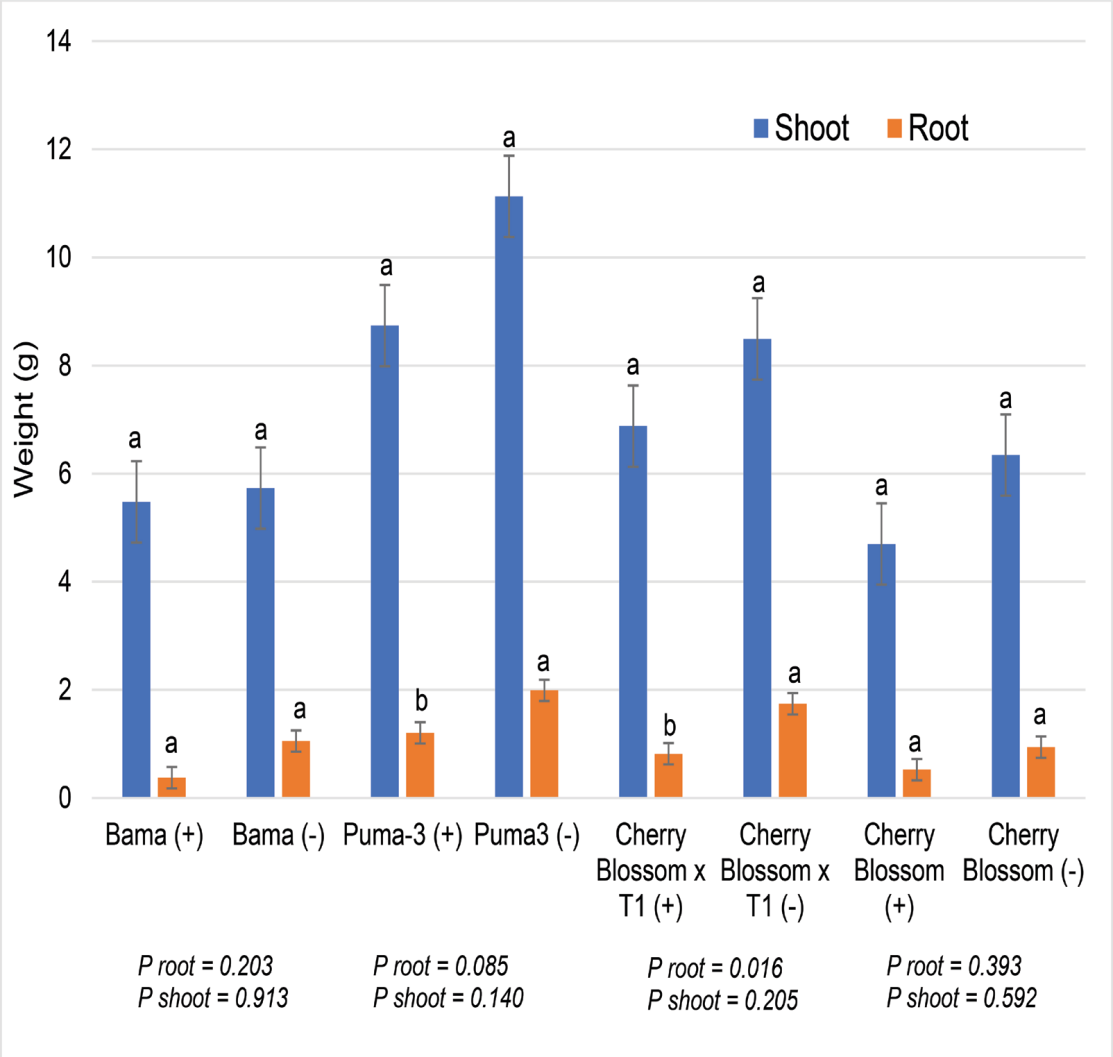
Hemp plant dry weights (g) for RKN inoculated (+) and uninoculated (−) plants with two CBD and two Chinese fiber hemp cultivars. Factor levels not connected by the same letter are significantly different according to Tukey’s HSD with *P* ≤ 0.05 (Trial 3).

Germination of cultivar Yuma-2 was very poor, and no further data were collected for this cultivar. There was no difference in root gall rating between cultivars (GI averaging 1.5 for hemp cultivars as compared to GI = 4.0 for cucumber). Overall, RKN reproduction in this trial was low (less than 1, as compared to 3.5 for cucumber) (Table 4). Just as in trial 1, there was no significant interaction in plant height between nematode-inoculated and uninoculated plants. Plant height was significantly greater for the fiber cultivars compared to the CBD cultivars (data not given). No difference in biomass weight was noted between cultivars, and while nematode-inoculated pots had numerically lower biomass weight for all cultivars, a significant reduction from nematodes was noted only for the root weight of cultivar Puma and cultivar Cherry Blossom T1 (Fig. 4).

**Table 4:** Nematode reproduction on two Chinese fiber and two CBD hemp cultivars after 60 days with a mixed population of *M. incognita* and *M. javanica* (Trial 3).

	**Gall Index (0–10)**	**Eggs/Whole Root**	**Eggs/g of Dry Root**	**J2s/pot**	**Reproduction Factor (Rf)**
Bama	1.8	580	250	1,350	0.193
Puma-3	1.8	250	220	3,320	0.357
Cherry Blossom × T1	1.4	280	240	3,470	0.376
Cherry Blossom	1.2	260	190	2,740	0.299
Cucumber^*^	4.0	27,520	-	8,298	3.58
*P* value	0.633	0.341	0.624	0.657	0.694

Gall index scale: 0–10; 0= no galls, 10= 100% galls. Eggs and J2s rounded to nearest ten. Reproduction factor (Rf = Pf/Pi) where Pf is total number of J2s and eggs after 60 days and Pi is 10,000 RKN eggs. Factor levels not connected by the same letter are significantly different according to Tukey’s HSD with *P* ≤ 0.05; cucumber data are given as comparison and were not included in the analysis.

### Trial 4: Evaluation of two US CBG Cultivars in naturally RKN-Infested Soil, with and without Nematicide

Both CBG hemp cultivars, cultivar Gold and cultivar Panacea, showed significant root galling and high nematode reproduction (Table 5), but these factors were lower in cultivar Panacea (GI 5.2) compared to cultivar Gold (GI 6.8). The tomato control averaged 8.2. The final population (Pf) was significantly (*P* = 0.0002) higher in cultivar Gold (which was similar to tomato; 253,600 eggs/root system, 30,620 J2/pot) than in cultivar Panacea (113,640 eggs/root, 14,240 J2/pot) (Table 5). Fluopyram application at planting reduced root galls and nematode reproduction by more than half for both hemp cultivars and tomatoes (Table 5).

**Table 5:** Nematode reproduction on two cannabigerol (CBG) hemp cultivars after 60 days with a mixed natural field population of *M. incognita* and *M. javanica* with and without nematicide (V) application (Trial 4).

	**Gall Index**	**Total Eggs/Root**	**Eggs/g Dry Root Wt.**	**J2s/Pot**	**Pf**
Gold	6.8b	253,600	192,620a	30,620b	284,230ab
Gold (V)	3.4de	82,980	68,710ab	19,980b	102,960b
Panacea	5.2c	113,640	47,430b	14,240b	127,880b
Panacea (V)	2.3e	47,100	24,400b	9,040b	44,910b
Tomato	8.2a	235,600	122,800ab	65,540a	301,140a
Tomato (V)	4.4cd	184,640	103,280ab	23,270b	207,910b
*P* value	< 0.0001	0.099	0.041	< 0.0001	0.0002

Gall index scale: 0–10; 0= no galls, 10= 100% galls. Eggs and J2s rounded to nearest ten. Pf is total number of J2s and eggs after 60 days. V = Velum (a.i. fluopyram). Factor levels not connected by the same letter are significantly different according to Tukey’s HSD with *P* ≤ 0.05.

**Figure 5:**
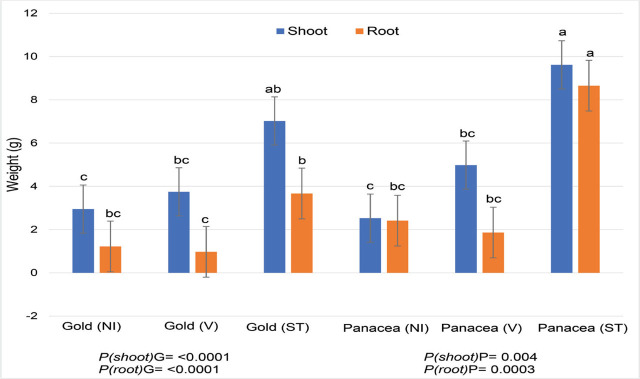
Hemp plant dry weights (g) for two cannabigerol (CBG) hemp cultivars and a nematicide in naturally RKN-infested soil. P values (*P* ≤ 0.05) represent significant differences between cultivars by treatment (PG = Gold, PP= Panacea; NA = naturally infested soil, V = Velum, ST = steamed soil.). Velum was applied at 0.48 kg a.i./Ha. Factor levels not connected by the same letter are significantly different according to Tukey’s HSD with *P* ≤ 0.05 (Trial 4).

**Figure 6:**
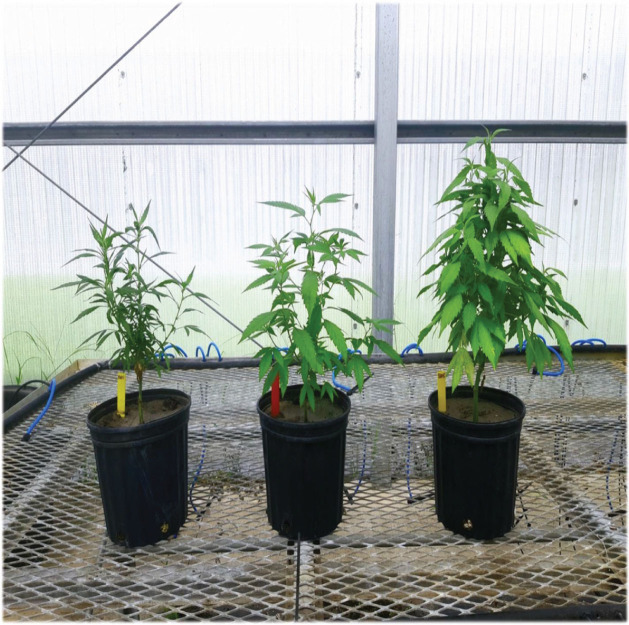
*Cannabis sativa* (cv. Panacea) 60 days after planting in RKN-infested field soil. (Left) naturally infested soil, (middle) nematicide-treated (fluopyram) soil, (right) steamed soil (Photo J. Coburn).

Overall, plants were short due to the transplants flowering only a few weeks after transplanting. Fluopyram increased plant height in cultivar Gold but not in cultivar Panacea (data not given). In terms of biomass weight, fluopyram had a greater positive impact on plant shoot weight of cultivar Panacea (50% increase) as compared to cultivar Gold, but root weights were not impacted by fluopyram (Fig. 5). Steaming soil prior to transplanting had a much greater effect than nematicide application on plant growth (Fig. 6). When planted in steamed field soil, plants of both cultivars nearly doubled in shoot weight as compared to when treated with the nematicide (Fig. 5). Dry root weights for both CBG cultivars in steamed soil increased four-fold when compared with fluopyram-treated plants (Fig. 5).

### Trial 5: Evaluation of Two Hemp Cultivars Against *M. hapla* and *M. enterolobii*

Nematode reproduction was low for both nematodes and for both hemp cultivars (Eletta Campana and Cherry Blossom × T1). Significant differences were noted between the two cultivars and nematode species (Table 6). In the case of *M. enterolobii* (*Me*), cultivar Cherry Blossom × T1 showed a GI of 1.4, and cultivar Eletta Campana a GI of 2.0. The reproduction factor for cultivar Cherry Blossom × T1 with *Me* was 0.348, while cultivar Eletta Campana had an Rf = 1.759. The cucumber control with *Me* showed a GI of 3.8 and Rf = 3.189, almost double that of both hemp cultivars. When inoculated with *M. hapla* (*Mh*), Cherry Blossom × T1 had a GI of 1.8, and Eletta Campana had a GI of 1.0. Reproduction of *Mh* was similarly low for both cultivars with an average Rf = 0.770. The cucumber control had a GI of 3.8, but a similarly low Rf = 0.638 (Table 6).

**Table 6:** Nematode reproduction of *M. enterolobii* and *M. hapla* on a CBD and fiber cultivar after 60 days (Trial 5).

	**Gall Index (0–10)**	**Eggs/Whole Root**	**Eggs/g of Dry Root**	**J2s/pot**	**Reproduction Factor (Rf)**
CBD - Cherry Blossom × T1 (+ Me)	1.4b	2,600	1,710	770b	0.338
Fiber - Eletta Campana (+ Me)	2.0b	16,300	7,475	1,300b	1.759
Cucumber (+ Me)	3.8a	27,030	16,850	4,860a	3.189
*P* value	0.004	0.188	0.187	0.046	0.158
CBD - Cherry Blossom × T1 (+ Mh)	1.8b	3,470a	1,490a	4,400	0.787
Fiber - Eletta Campana (+ Mh)	1.0b	1,640b	790ab	5,860	0.750
Cucumber (+ Mh)	3.8a	500b	130b	5,880	0.638
*P* value	< 0.0001	0.003	0.025	0.773	0.871

Gall index scale: 0–10; 0= no galls, 10= 100% galls. Eggs and J2s rounded to nearest ten. Reproduction factor (Rf = Pf/Pi) where Pf is total number of J2s and eggs after 60 days and Pi is 10,000 RKN eggs. Factor levels not connected by the same letter are significantly different according to Tukey’s HSD with *P* ≤ 0.05.

**Figure 7:**
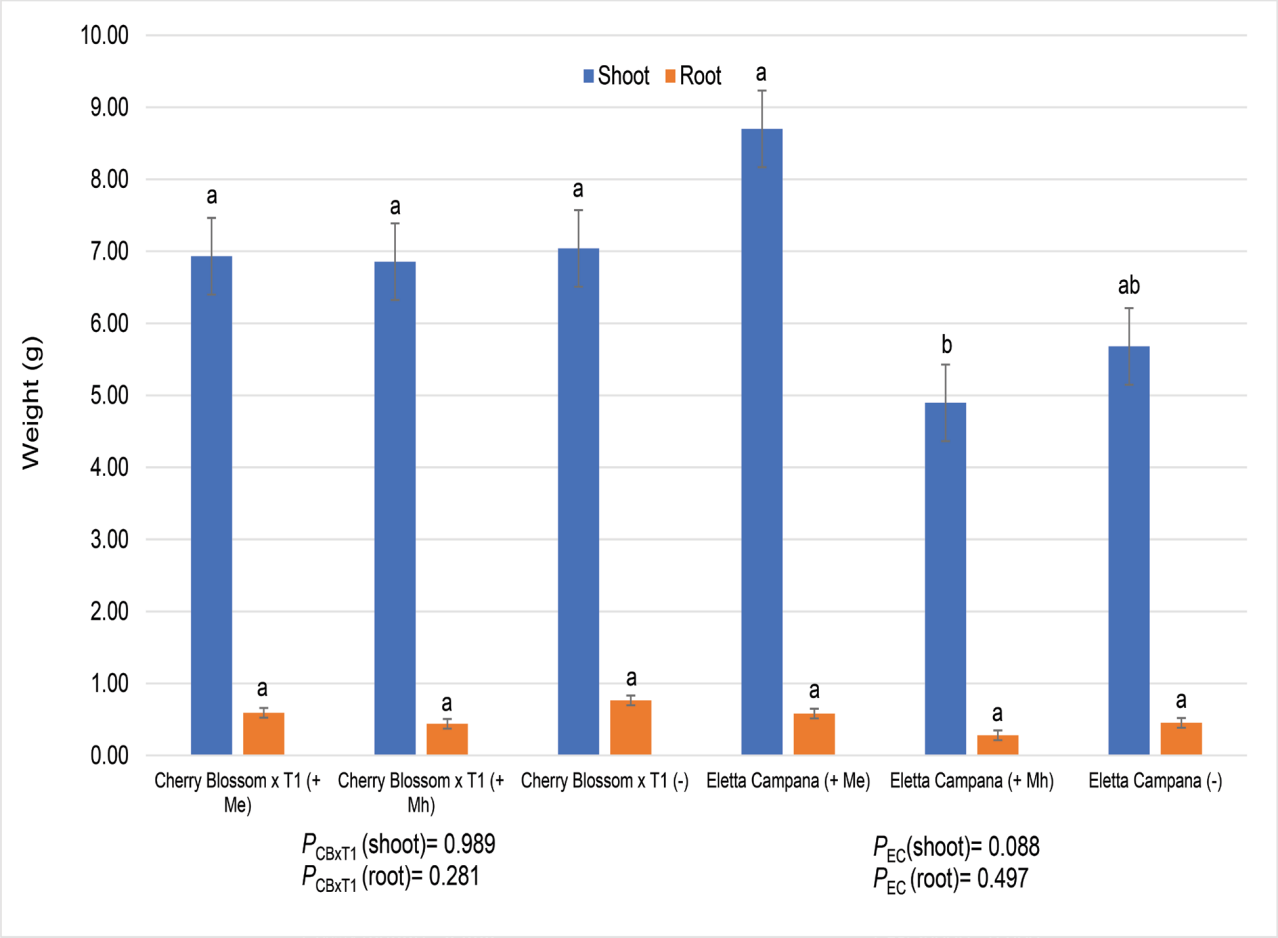
Hemp plant dry weights (g) two hemp cultivars (Eletta Campana = fiber, Cherry Blossom × T1 = CBD) exposed to two RKN species, *M. enterolobii* and *M. hapla*. Factor levels not connected by the same letter are significantly different according to Tukey’s HSD where *P* ≤ 0.05. PCBxT1 = Cherry Blossom × T1, PEC = Eletta (Trial 5).

Plant height of cultivar Cherry Blossom × T1 was not negatively impacted by either RKN species with an average height of 50 cm, but for cultivar Eletta Campana, plant height was higher when inoculated with *Me* as compared to *Mh* (data not given). The same was noted for dry shoot weights, with no difference noted for cultivar Cherry Blossom × T1 but higher for cultivar Eletta Campana when inoculated with *Me* when compared to *Mh* (Fig. 7).

## Discussion

All hemp cultivars were good hosts for RKN based on root gall symptoms and nematode reproduction. Root gall ratings were generally less than those noted on the cucumber or tomato controls, but reproduction was often similarly high. The mixed population of *M. javanica* and *M. incognita* (trials 1, 2, and 4) reproduced rapidly on all tested cultivars (fiber, dual, CBD, and CBG). The same population showed fewer root galls and lower reproduction in trial 3. This effect probably was related to the time of the year the experiment was conducted (winter), as cucumber control plants in these trials also had fewer root galls and lower nematode reproduction. Lower greenhouse temperatures during winter (20–21 °C as compared to 25–26 °C in spring-summer) and shorter day length are probably the main reason for this. The same was noted for trial 5 (pure populations of *M. enterolobii* and *M. hapla*), which was also done during winter. Trials 1, 2, and 4 were done during spring-summer, and all hemp cultivars in those trials showed high root-knot infection and reproduction. Tyler (1933) reported an optimum temperature for thermophilic root-knot nematodes of 28 °C, and several other authors have reported slower development of *M. incognita* and *M. javanica* at lower temperatures, such as those observed in our winter trials (Davide and Triantaphyllou, 1967; Tzortzakakis and Trudgill, 2005; Davila-Negron and Dickson, 2013). In the case of *M. hapla*, the northern root-knot nematode (trial 5), which is better adapted to cooler temperatures (Vrain et al., 1978), lower temperatures would not explain the low reproduction. Possibly, the specific population of *M. hapla*, which originates from a local strawberry field, was not particularly virulent. Previous greenhouse trials in our lab with *M. hapla* also often failed to show high nematode reproduction on crops other than strawberries (personal observation). In addition to lower temperatures, slower plant growth in winter because of shorter day lengths could also have affected nematode infectivity and reproduction. Especially for daylength-sensitive plants like hemp, plant growth is significantly impacted by photoperiod, which was clearly shown by reduced hemp plant weights when trials were done during winter.

Our results confirm several recent reports from the US and China showing that hemp can be a good host for various RKN, including *M. incognita, M. hapla,* and *M. enterolobii* (Song et al., 2017; Kotcon et al., 2018; Ren et al., 2020; Lawaju et al., 2021; Bernard et al., 2022; Coburn et al., 2022).

No clear differences among hemp cultivars in nematode symptoms and host status were seen, except in experiment 2, where cultivar Cherr y Blossom had less visible root galls than other cultivars, although reproduction was similar. Differences in nematode reproduction have been reported on hemp cultivars with *M. incognita* and *M. hapla* (Kotcon et al., 2018), and resistance against *M. incognita* was recently reported in the CBD cultivar Wife (Hansen et al., 2020). Root-knot resistance would be a very useful trait if hemp is to become a major crop in Florida and other southeastern US states, where root-knot nematodes are among the most widespread and damaging soilborne pathogens.

The main differences observed in plant height and biomass between cultivars were due to the physiology of the plants. The Chinese fiber cultivars are naturally tall and slender, the European fiber and dual-use cultivars are slightly shorter than the fiber hemp cultivars, and CBD plants are short and bushy. In most trials, no negative effects of root knot on hemp shoot growth were observed, except for a negative effect on dry root weight from the mixed population of *M. javanica* and *M. incognita* for the European fiber/dual-use cultivars Helena, Fibranova, and Eletta Campana, the Chinese fiber cultivar Puma-3, and the US CBD cultivar Cherry Blossom × T1. The exception was in trial 4, where naturally infested soil (from a local cucumber field) was used. In this trial, growth of two CBG cultivars, Gold and Panacea, was significantly reduced, as was shown by visibly improved growth when a nematicide was applied, and especially when soil was pasteurized. This shows that under natural conditions in the field, hemp growth could be greatly reduced by RKN. Most greenhouse trials are conducted by inoculating nematodes into pasteurized soil, which is a good method to evaluate nematode host status, but it may underestimate actual nematode damage potential under natural conditions.

Considering the importance of RKN in Florida, it is essential to evaluate any new crop that is introduced in the state for its susceptibility and host status to this nematode. Hemp or *Cannabis sativa* is one of these new crops, and all tested hemp cultivars in our trials, regardless of use (fiber, dual, CBD) or origin (Europe, China, US), were good hosts for local RKN populations (*M. incognita* + *M. javanica*, *M. enterolobii,* and to a lesser extent *M. hapla*). All these *Meloidogyne* species are common in Florida, and often, fields are infested with multiple species (Riva et al., 2022).

Our study provides a first assessment of the effect of different root-knot nematodes on a range of *C. sativa* cultivars. This knowledge is important for first-time farmers who want to grow hemp, as well as for established farmers who are considering using hemp in their current production system as a rotation crop.

## References

[j_jofnem-2024-0003_ref_001] Atakan Z (2012). Cannabis, a complex plant: Different compounds and different effects on individuals. Therapeutic Advances in Psychopharmacology.

[j_jofnem-2024-0003_ref_002] Bernard E.C., Chaffin A.G., Gwinn K.D. (2022). Review of nematode interactions with hemp (Cannabis sativa). Journal of Nematology.

[j_jofnem-2024-0003_ref_003] Bridge J., Page S.L.J. (1980). Estimation of root-knot nematode infestation levels on roots using a rating chart. Tropical Pest Management.

[j_jofnem-2024-0003_ref_004] Cherney J, Small E. (2016). Industrial hemp in North America: Production, politics and potential. Agronomy (Basel).

[j_jofnem-2024-0003_ref_005] Coburn J.D., Moreira D., Freeman J., Gu M., Bui H.X., Desaeger J.A. (2022). First report of Meloidogyne incognita infecting Cannabis sativa in Florida, USA. Nematropica.

[j_jofnem-2024-0003_ref_006] Davide R.G., Triantaphyllou A.C. (1967). Influence of the environment on development and sex differentiation of root-knot nematodes. Nematologica.

[j_jofnem-2024-0003_ref_007] Davila-Negron M., Dickson D. (2013). Comparative thermal time requirements for development of Meloidogyne arenaria, M. incognita, and M. javanica, at constant temperatures. Nematropica.

[j_jofnem-2024-0003_ref_008] Desaeger J. (2018a). Meloidogyne hapla, the northern root-knot nematode, in Florida strawberries and associated double-cropped vegetables.

[j_jofnem-2024-0003_ref_009] Desaeger J. (2018b). Nematodes parasitizing hops in Florida.

[j_jofnem-2024-0003_ref_010] Fike J.H. (2016). Industrial Hemp: Renewed Opportunities for an Ancient Crop. Critical Reviews in Plant Sciences.

[j_jofnem-2024-0003_ref_011] Grinspoon P. (2018). Cannabidiol (CBD)—what we know and what we don’t. Harvard Health Blog.

[j_jofnem-2024-0003_ref_012] Hansen Z., Gwinn K., Hale F., Kelly H., Stewart S. (2020). Hemp disease and pest management.

[j_jofnem-2024-0003_ref_013] Hussey R. S., Barker K. R. (1973). Comparison of methods of collecting inocula of Meloidogyne spp., including a new technique. Plant Disease.

[j_jofnem-2024-0003_ref_014] Kotcon J.B., Wheeler K., Cline R., Carter S.L. (2018). Susceptibility and yield loss relationships of Meloidogyne hapla and M. incognita infecting Cannabis sativa. [Abstract]. Journal of Nematology.

[j_jofnem-2024-0003_ref_015] Lawaju B.R., Groover W., Kelton J., Conner K., Sikora E., Lawrence K.S. (2021). First report of Meloidogyne incognita infecting Cannabis sativa in Alabama. Journal of Nematology.

[j_jofnem-2024-0003_ref_016] Ren Z., Chen X., Luan M., Guo B., Song Z. (2020). First report of Meloidogyne enterolobii on industrial hemp (Cannabis sativa) in China. Plant Disease.

[j_jofnem-2024-0003_ref_017] Riva G., Bui H. P., Gu M.P., Desaeger J. (2022). Molecular detection and distribution of root-knot nematode species in Florida, USA.

[j_jofnem-2024-0003_ref_018] Sasser J. N. (1980). Root-knot nematodes: A global menace to crop production. Plant Disease.

[j_jofnem-2024-0003_ref_019] Sasser J. N., Carter C. C., Sasser J. N., Carter C.C. (1985). Overview of the international Meloidogyne project 1975–1984. An advanced treatise on Meloidogyne: Vol. I. Biology and control.

[j_jofnem-2024-0003_ref_020] Song Z.Q., Cheng F.X., Zhang D.Y., Liu Y., Chen X.W. (2017). First report of Meloidogyne javanica infecting hemp (Cannabis sativa) in China. Plant Diseases.

[j_jofnem-2024-0003_ref_021] Thies J.A., Merrill S.B., Corley E.L. (2002). Red food coloring stain: New, safer procedures for staining nematodes in roots and egg masses on root surfaces. Journal of Nematology.

[j_jofnem-2024-0003_ref_022] Tyler J. (1933). Development of the root-knot nematode as affected by temperature. Hilgardia.

[j_jofnem-2024-0003_ref_023] Tzortzakakis E.A., Trudgill D.L. (2005). A comparative study of the thermal time requirements for embryogenesis in Meloidogyne javanica and M. incognita. Nematology.

[j_jofnem-2024-0003_ref_024] Viglierchio D.R., Schmitt R.V. (1983). On the methodology of nematode extraction from field samples: Baermann funnel modifications. Journal of Nematology.

[j_jofnem-2024-0003_ref_025] Vrain T. C., Barker K.R., Holtzman G.I. (1978). Influence of low temperature on rate of development of Meloidogyne incognita and M. hapla larvae. Journal of Nematology.

[j_jofnem-2024-0003_ref_026] Williams D. W., Mundell R. (2015). An introduction to industrial hemp and hemp agronomy.

